# Treatment and survival of osteosarcoma and Ewing sarcoma of the skull: a SEER database analysis

**DOI:** 10.1007/s00701-018-3754-y

**Published:** 2018-12-21

**Authors:** Enrico Martin, Joeky T. Senders, P. Valerie ter Wengel, Timothy R. Smith, Marike L. D. Broekman

**Affiliations:** 1 Computational Neurosciences Outcomes Center, Department of Neurosurgery, Brigham and Women’s Hospital, Harvard Medical School, 60 Fenwood Road, Boston, MA 02115 USA; 2 Department of Plastic and Reconstructive Surgery, University Medical Center Utrecht, Utrecht, The Netherlands; 3 Department of Neurosurgery, Brain Center Rudolf Magnus, University Medical Center Utrecht, Utrecht, The Netherlands; 4 Department of Neurosurgery, Slotervaart Ziekenhuis Amsterdam, Amsterdam, The Netherlands; 5 Department of Neurosurgery, VU Medical Center Amsterdam, Amsterdam, The Netherlands; 6 Department of Neurosurgery, Leiden University Medical Center, PO Box 9600, 2300 RC Leiden, The Netherlands; 7 Department of Neurosurgery, Haaglanden Medical Center, The Hague, The Netherlands

**Keywords:** Ewing sarcoma, Osteosarcoma, Cranial, Skull tumor, SEER

## Abstract

**Background:**

Common primary bone tumors include osteosarcomas (OSC) and Ewing sarcomas (EWS). The skull is a rare site, and literature about their treatment and survival is scarce. Using the Surveillance, Epidemiology, and End Results (SEER) database, this study aims to assess the treatment and survival of skull OSC and skull EWS, as well as predictors for survival.

**Methods:**

Skull OSC and EWS cases were obtained from the SEER database. Patient and tumor characteristics, treatment modalities, and survival were extracted. Overall survival (OS) was assessed using multivariable Cox proportional hazard regression stratified by tumor histology. Kaplan-Meier curves were constructed for OS comparing OSC and EWS, as well as histological subtypes in OSC.

**Results:**

A total of 321 skull OSC and 80 skull EWS patients were registered from 1973 to 2013. EWS was more common in younger patients (*p* < 0.001). Resection was the predominant treatment strategy (80.1%), frequently in combination with adjuvant radiotherapy (30.4%). The 5-year survival rate varied significantly between OSC and EWS (51.0% versus 68.5%, *p* = 0.02). Kaplan-Meier curves show that EWS had a significantly better survival compared to OSC. Comparing histological subtypes of skull OSC, chondroblastic OSC had the best OS, Paget OSC the worst. Older age, tumor advancement, no surgical treatment, and the use of radiotherapy were identified as independent predictors of decreased OS in skull OSC.

**Conclusion:**

Overall prognosis is better for EWS compared to OSC. Chondroblastic OSC have the best overall survival, while OSC associated with Paget’s disease of the bone has the poorest overall survival.

## Introduction

Primary malignant bone tumors of the skull are rare entities. Osteosarcomas (OSC) and Ewing sarcomas (EWS) are common primary bone tumors which can also affect the skull. However, in comparison to skull base chordoma and chondrosarcoma, they are rare entities. OSC are the most common malignant tumors of the bone, with a peak incidence in adolescence during the growth spurt [27]. They can arise after radiotherapy, in Paget’s disease of the bone, but also in several hereditary diseases [6, 27]. As in other sites of origin, skull OSC can also arise after trauma [23, 29]. It is estimated that 1.6% of all OSC are present in the skull [28]. EWS are the second most common primary bone malignancies in children specifically [45]. They typically occur in children and adolescents, but also in adults [27, 45]. The reported incidence of primary cranial EWS is approximately 1% of all possible sites of origin [2, 16, 44].

To date, literature on OSC and EWS primarily arising from the skull has been limited to case reports or small case series [14, 22, 23, 39, 40]. This is primarily due to the low frequency of this tumor site. Consequently, survival and ideal treatment of these rare tumors is not very well known. Moreover, studies have, yet, neither been able to investigate possible predictors of worse survival, nor been able to stratify results for histological subtypes of skull OSC. The Surveillance, Epidemiology, and End Results (SEER) program is a cancer registry that collects data from 18 geographic areas across the USA, currently encompassing approximately 28% of its population [34]. It provides a means of assessing possible predictive factors of survival and treatment strategies for rare tumors such as primary osseous malignancies of the skull. This study aims to evaluate the treatment and survival of skull OSC and skull EWS as well as determinants for decreased survival. Also, skull OSC will be stratified by histological subtype to investigate possible differences in survival.

## Methods

### Data source

Data were obtained from the SEER program from 1973 to 2013. The International Classification of Disease for Oncology (ICD-O-3) histology codes were used to identify cases. OSC (ICD-O-3: 9180/3, 9181/3, 9182/3, 9183/3, 9184/3, 9185/3, 9186/3, 9193/3), and EWS (9260/3) arising from the skull or intracranially were selected (C41.0, C70.0, C71.1, C71.2). Over time, ICD-codes have been updated according to new classifications by trained clinical reviewers. Parosteal osteosarcomas are low-grade tumors and were therefore not included in our analysis. Our Institutional Review Board has exempted the SEER program from review.

### Covariates

Covariates extracted for analysis were as follows: sex, age, or categorical age (≤ 18, 19–50, and ≥ 50 years), race (White, Black, Asian, and other), period of diagnosis (1973–1983, 1984–1993, 1994–2003, 2004–2013), tumor size, extent of surgery (no surgery, partial resection, gross total resection, surgery NOS, unknown status of surgery), radiotherapy (yes/no), and timing of radiotherapy to surgery (prior to, after, during, prior to, and after surgery). Extent of disease (EOD) was reclassified into one variable, as previously established in the literature, using both EOD and collaborative stage (CS) coding methods, into local, locally advanced, and metastatic [35]. Surgical procedures were coded differently in SEER over time. In order to evaluate surgery from all time periods, a single variable was constructed: no surgery (< 1998 codes: 00, 01; for 1998 +: 00), partial resection (< 1998: 02, 10, 20, 28; 1998 +: 15, 19, 25, 26), gross total resection (GTR, < 1998: 30, 38, 40, 48; 1998 +: 30, 40, 41, 42, 55), surgery not otherwise specified (< 1998: 60, 68, 90; 1998 +: 90), and unknown status of surgery (< 1998: 09, 80).

### Statistical analysis

Baseline characteristics of OSC and EWS were analyzed using descriptive statistics. Only primary tumors were included in the survival analyses. Multivariable Cox proportional hazard analyses were performed stratified for OSC and EWS to evaluate possible independent predictors of overall survival (OS). Variables of interest were identified through univariable analysis and included in multivariable analysis when *p* values were < 0.20 [7]. The rule of one predictor per 10 events was used to avoid overfitting of the model. *p* values < 0.05 were considered statistically significant. Missing values were imputed using a validated random forest algorithm from the MICE package in R, which has been shown to yield the highest reliability for data imputation [47, 48]. Schoenfeld residuals were assessed to check if variables were independent from time. All variables included in the final models had a *p* value > 0.05. Global *p* values of the osteosarcoma model and Ewing sarcoma model were 0.12 and 0.33 respectively. Kaplan-Meier curves for OS were constructed for EWS and OSC. Subsequent analyses were performed for skull OSC subtypes with 10 or more registered; remaining tumors were grouped into the “other” group. Statistical analyses and figures were conducted using R version 3.3.3 (R Core Team 2016).

## Results

### Patient population

**Fig. 1:**
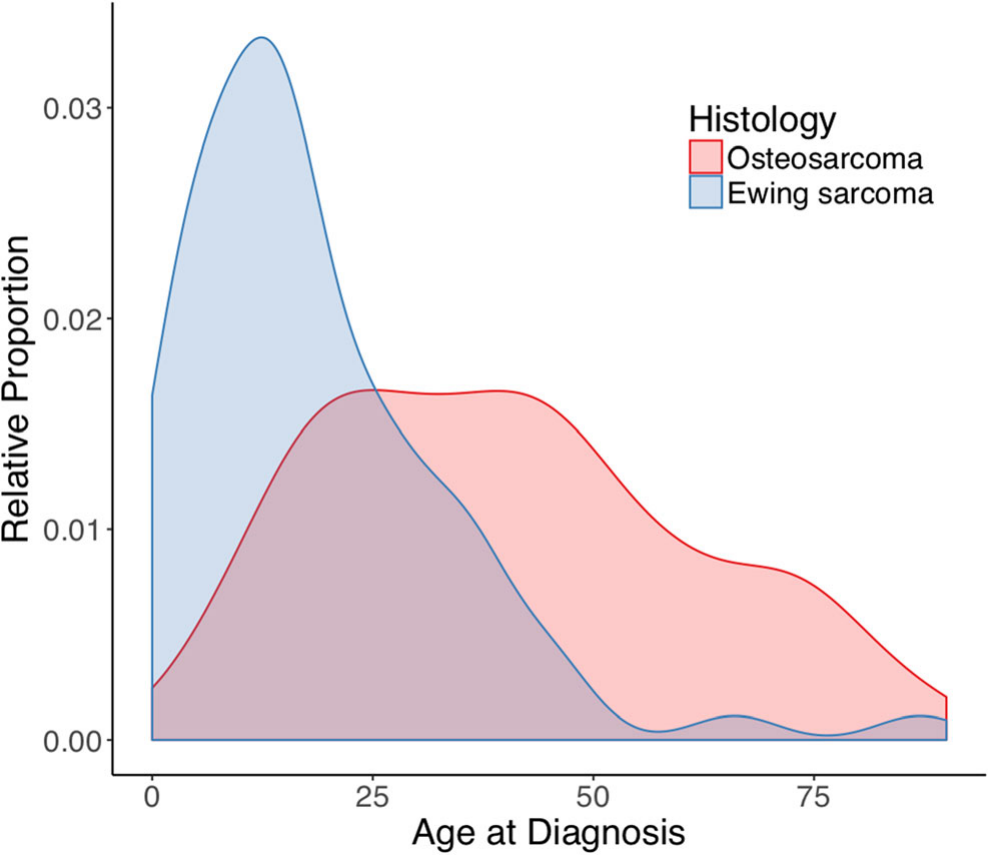
Relative age distribution of osteosarcoma and Ewing sarcoma of the skull

A total of 401 patients were identified in the SEER database: 321 patients with skull osteosarcomas (OSC) (80.0%) and 80 patients with skull Ewing sarcomas (EWS) (20.0%, Table 1). The median age was 32 years (IQR 17–52) overall, but 16 years (IQR 8–29) in EWS and 38 years (IQR 21–57) in OSC (*p* < 0.001, Fig. 1). A slight majority of all patients was male (52.4%), and most commonly White (81.5%), without significant differences between EWS and OSC. Most tumors were locoregional (57.9%) and metastases were infrequent at time of diagnosis: 4.7% and 2.5% for OSC and EWS respectively (*p* > 0.05). There were no differences in tumor size and median tumor size was 45.0 mm (IQR 28.0–51.0). EWS had statistically significant different 5-year survival rates compared OSC; 68.5% and 51.5% respectively (*p* = 0.02).

**Table 1:** Patient and treatment demographics in skull osteosarcomas and Ewing sarcomas

Characteristic	Definition	Total (*N* = 401)	Osteosarcoma (*N* = 321)	Ewing Sarcoma (*N* = 80)	*P*
Age	≤ 18	111 (27.7)	64 (19.9)	47 (58.8)	*< 0.001*
	19–50	181 (45.1)	153 (47.7)	28 (35.0)	
	50 +	109 (27.2)	104 (32.4)	5 (6.3)	
	Median (year)	32	38	16	*< 0.001*
	IQR	17–52	21–57	8–29	
Sex	Female	191 (47.6)	152 (47.2)	40 (50.0)	0.64
	Male	210 (52.4)	170 (52.8)	40 (50.0)	
Race	White	327 (81.5)	257 (80.1)	70 (87.5)	0.30
	Black	48 (12.0)	43 (13.4)	5 (6.3)	
	Asian and other	24 (6.0)	19 (5.9)	5 (6.3)	
	Unknown	2 (0.5)	2 (0.6)	0 (0.0)	
Extent of disease	Local	105 (26.2)	86 (26.8)	19 (26.2)	0.72
	Locoregional	232 (57.9)	182 (56.7)	50 (62.5)	
	Metastasized	17 (4.2)	15 (4.7)	2 (2.5)	
	Unknown	47 (11.7)	38 (11.8)	9 (11.3)	
Tumor size	≤ 50 mm	179 (44.6)	145 (45.2)	34 (42.5)	0.78
	> 50 mm	103 (25.7)	80 (24.9)	23 (28.8)	
	Missing	119 (29.7)	96 (29.9)	23 (29.7)	
	Median (mm)	45.0	45.0	50.0	0.53
	IQR (mm)	35.0–60.0	35.0–60.0	40.0–60.0	
Treatment modality	Sx only	192 (47.9)	177 (55.1)	15 (18.8)	*< 0.001*
	Rx only	24 (6.0)	9 (2.8)	15 (18.8)	
	Sx and Rx	129 (32.2)	85 (26.5)	44 (55.0)	
	None	33 (8.2)	30 (9.3)	3 (3.8)	
	Unknown	23 (5.7)	20 (6.2)	3 (3.8)	
Extent of resection	No surgery	57 (14.2)	39 (12.1)	18 (22.5)	0.12
	Partial resection	138 (34.4)	109 (34.0)	29 (36.3)	
	GTR	121 (30.2)	101 (31.5)	20 (25.0)	
	Surgery NOS	71 (17.7)	61 (19.0)	10 (12.5)	
	Unknown	14 (3.5)	11 (3.4)	3 (3.8)	
Radiotherapy	No radiation	231 (57.6)	212 (66.0)	19 (23.8)	*< 0.001*
	Any form radiation	163 (40.1)	100 (31.2)	61 (76.3)	
	Unknown	9 (2.2)	9 (2.8)	0 (0.0)	
Radiotherapy sequence	No Rx or no Sx	271 (*NA*)	235 (*NA*)	36 (*NA*)	*< 0.001*
	Rx after Sx	122 (93.8)	79 (91.9)	43 (97.7)	
	Rx before Sx	6 (4.6)	5 (5.8)	1 (2.3)	
	Rx b/a Sx	0 (0.0)	0 (0.0)	0 (0.0)	
	Intraoperative Rx	2 (1.5)	2 (2.3)	0 (0.0)	
Time period	1973–1983	37 (9.2)	29 (9.0)	8 (10.0)	*0.001*
	1984–1993	49 (12.2)	46 (14.3)	3 (3.8)	
	1994–2003	138 (34.4)	119 (37.1)	19 (23.8)	
	2004–2013	177 (44.1)	127 (39.6)	50 (62.5)	
5-Y survival*	%	54.5%	51.0%	68.5%	*0.02*
10-Y survival**	%	37.4%	36.1%	44.4%	0.34

They are all significant at a *p*-value smaller than 0.05

*210 OS, 54 EWS eligible

**183 OS, 36 EWS eligible

*b/a* before and after, *IQR* interquartile range, *GTR* gross total resection, *mm* millimeters, *NOS* not otherwise specified, *Sx* surgery, *Rx* radiotherapy, *Y* year

### Treatment modalities

Most patients were only treated surgically (47.9%), with a combination of surgery and radiotherapy being the second most common treatment modality (32.2%, Table 1). Radiotherapy was more commonly administered in EWS, but in most cases also in combination with surgery. Both in EWS and in OSC, radiation was most commonly administered postoperatively (97.7% and 91.9% respectively). Extent of resection did not differ significantly between both tumors (*p* = 0.12). Partial resection was achieved in 41.8% and GTR in 36.7% of all cases.

### Overall survival in EWS and OSC

**Fig. 2:**
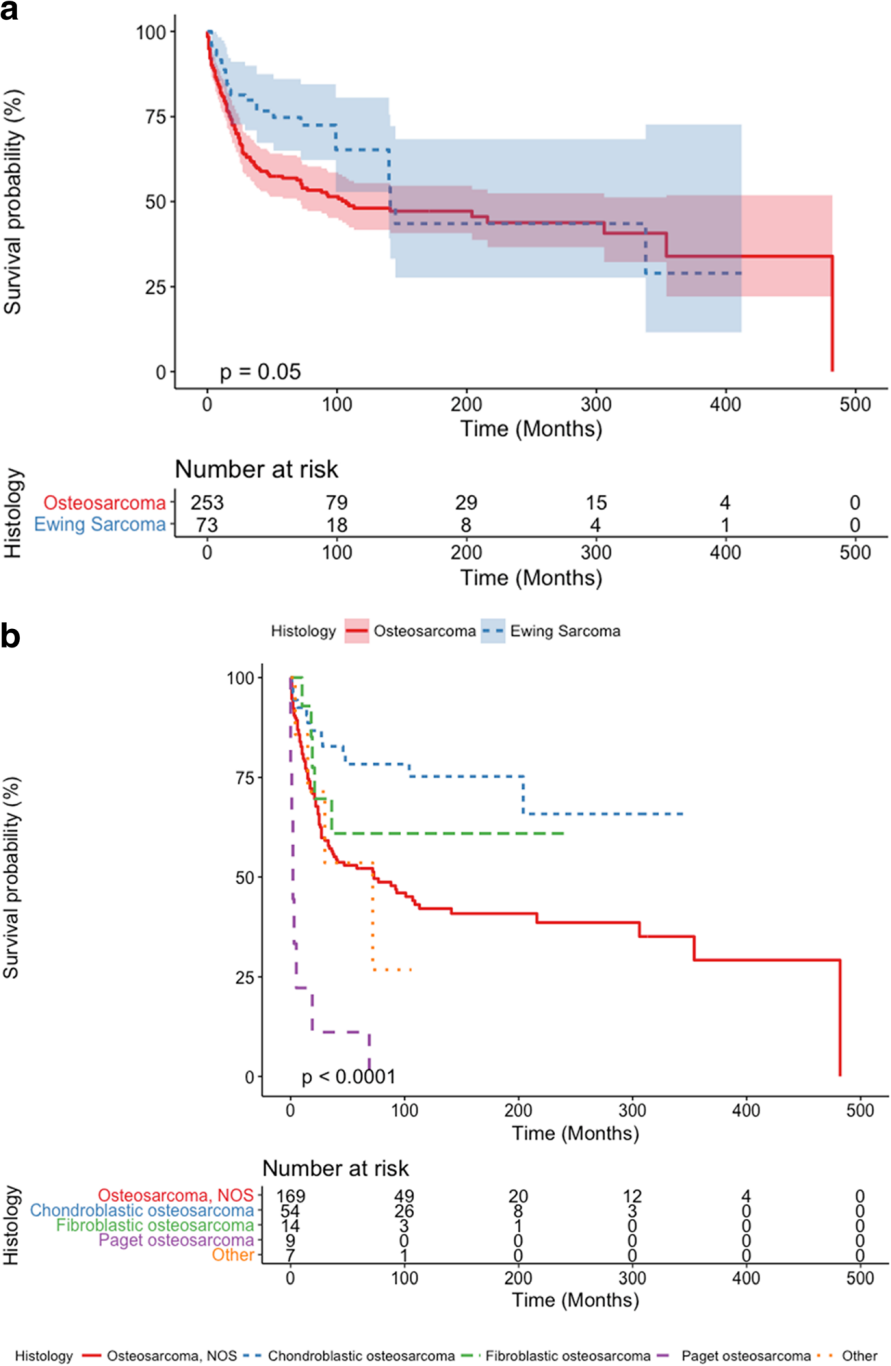
**a** Kaplan-Meier curves showing cumulative overall survival of osteosarcoma and Ewing sarcoma patients. **b** Kaplan-Meier curves showing cumulative overall survival in histological subtypes of osteosarcoma patients

**Fig. 3:**
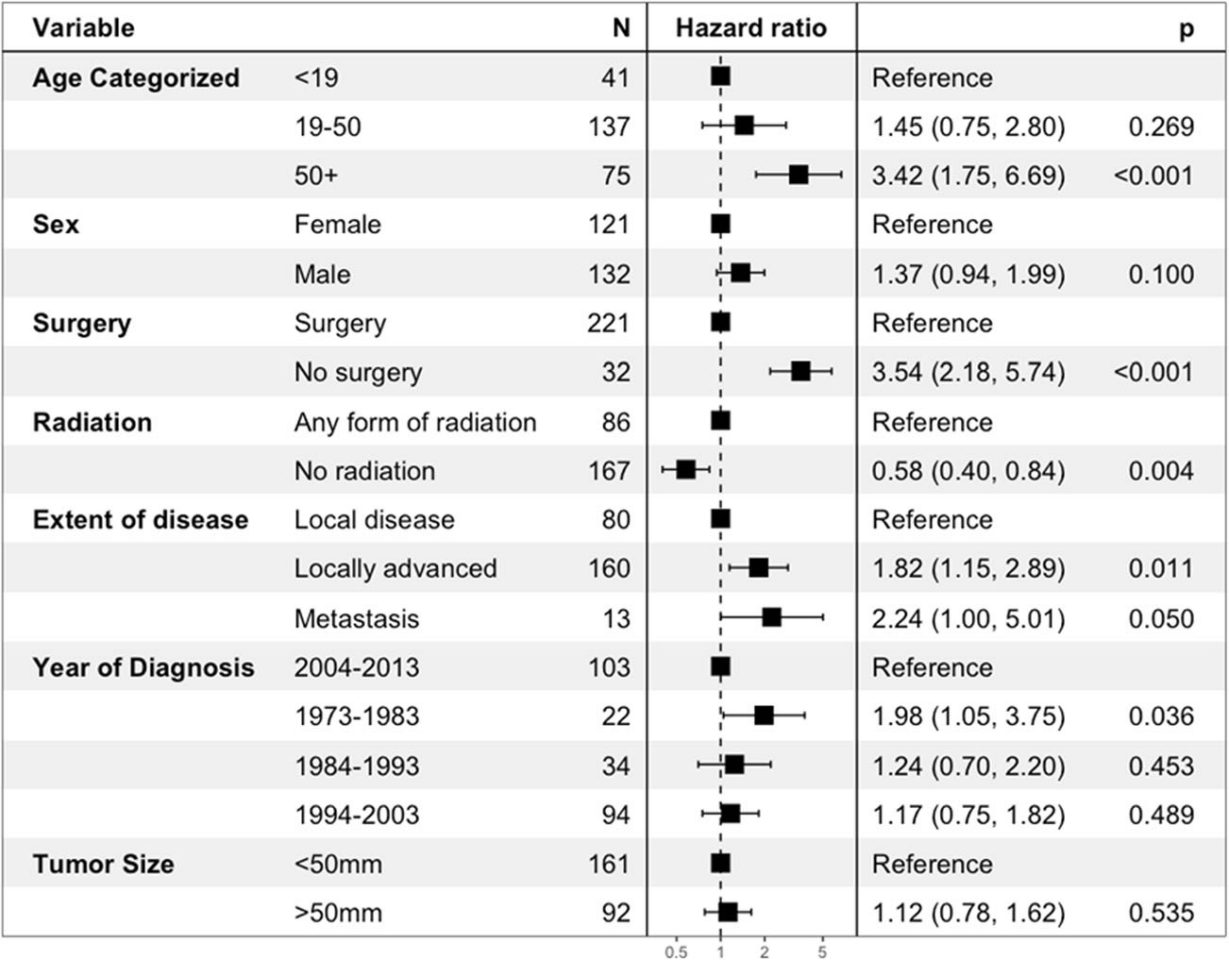
Multivariable Cox proportional hazard model for overall survival in osteosarcoma of the skull showing hazard ratios in a forest plot with 95% confidence intervals

**Fig. 4:**
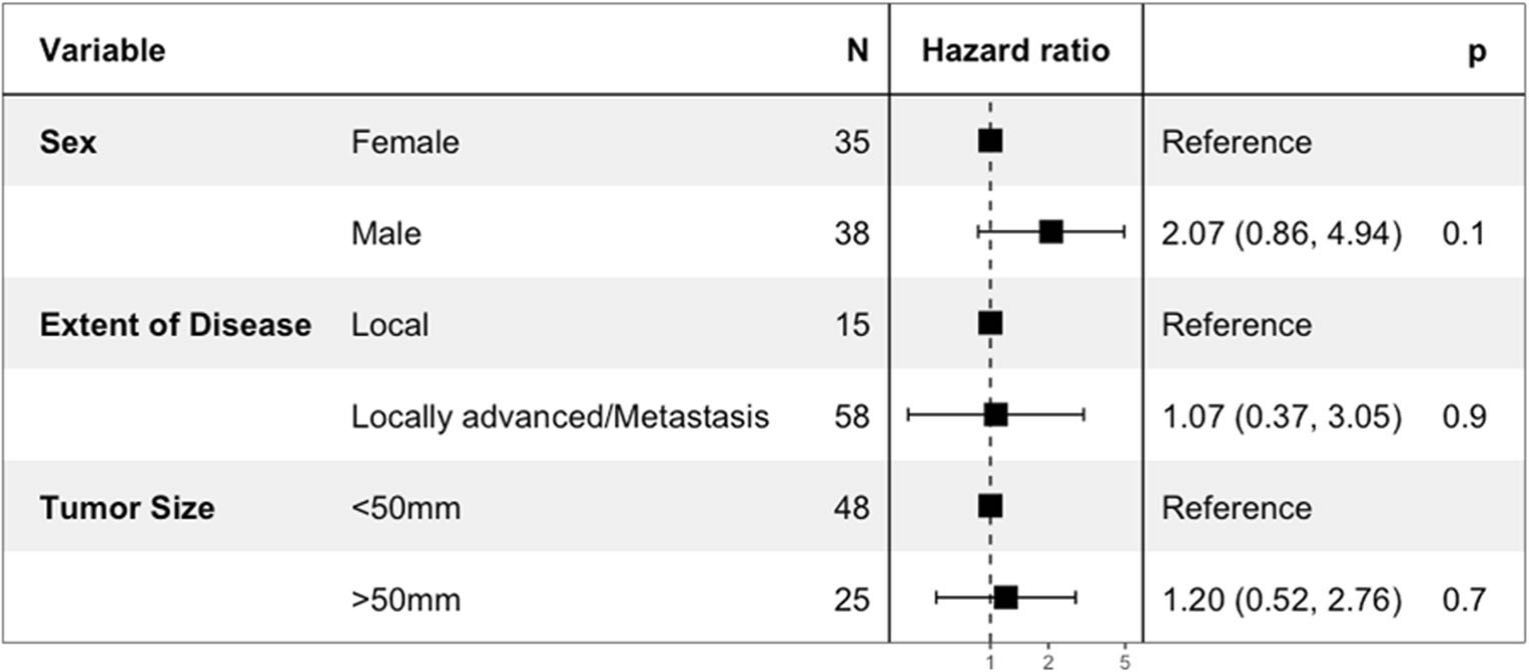
Multivariable Cox proportional hazard model for overall survival in Ewing sarcoma of the skull showing hazard ratios in a forest plot with 95% confidence intervals

EWS have a significantly better survival compared to OSC (Fig. 2a). In skull OSC, older age (≥ 50 years), no surgical treatment, advancement of disease, and early cases (1973–1983) were independent predictors of decreased overall survival after correction for sex and tumor size (Fig. 3). Patients that did not receive radiotherapy were independently correlated with superior OS. In skull EWS, no factors were identified that significantly affected OS in multivariate analyses (Fig. 4).

### Differences between osteosarcoma subtypes

There are several subtypes of OSC: osteoblastic, chondroblastic, fibroblastic, telangiectatic, small cell, low-grade central, parosteal, periosteal, high grade surface, and secondary including those arising in Paget’s disease of the bone [31]. The SEER database included 4 subtypes of skull OSC with more than 10 cases. These are 66 chondroblastic OSC, 14 fibroblastic OSC, 11 Paget OSC, and 222 OSC not otherwise specified. While most OSC arose in patients aged 19–50, those arising as a result of Paget’s disease were all in patients over 50 years (Table 2). Sex, treatment modalities, extent of disease, and tumor size did not differ significantly between these subtypes. However, 5- and 10-year survival rates significantly varied between the subtypes (*p* < 0.001). Kaplan-Meier curves showed that chondroblastic OSC have the best overall survival, while Paget osteosarcomas had a dismal prognosis (Fig. 2b). Overall differences were statistically significant (*p* < 0.001).

**Table 2:** Demographic differences per subtype of osteosarcoma

Characteristic	Definition	Osteosarcoma, NOS (*N* = 222)	Chondroblastic osteosarcoma (*N* = 66)	Fibroblastic osteosarcoma (*N* = 14)	Paget osteosarcoma (*N* = 11)	*P*
Age	≤ 18	45 (20.3)	15 (22.7)	2 (14.3)	0 (0.0)	< 0.001
	19–50	108 (48.6)	37 (56.1)	7 (50.0)	0 (0.0)	
	50 +	69 (31.1)	14 (21.2)	5 (35.7)	11 (100.0)	
Sex	Female	105 (47.3)	20 (45.5)	10 (71.4)	4 (36.4)	0.28
	Male	117 (52.7)	36 (54.5)	4 (28.6)	7 (63.6)	
Surgery	Yes	187 (84.2)	59 (89.4)	11 (78.6)	7 (63.6)	0.20
	No	25 (11.3)	7 (10.6)	3 (21.4)	3 (27.3)	
	Unknown	10 (4.5)	0 (0.0)	0 (0.0)	1 (9.1)	
Radiotherapy	No	145 (65.3)	48 (72.7)	9 (64.3)	6 (54.5)	0.62
	Yes	71 (32.0)	15 (22.7)	5 (35.7)	5 (45.5)	
	Unknown	6 (2.7)	3 (4.5)	0 (0.0)	0 (0.0)	
Extent of disease	Local	57 (25.7)	21 (31.8)	4 (28.6)	2 (18.2)	0.45
	Locoregional	123 (55.4)	40 (60.6)	8 (57.1)	5 (45.5)	
	Metastasized	13 (5.9)	1 (1.5)	0 (0.0)	1 (9.1)	
	Unknown	29 (13.1)	4 (6.1)	2 (14.3)	3 (27.3)	
Tumor size	≤ 50 mm	97 (43.7)	30 (45.5)	8 (57.1)	5 (45.5)	0.61
	> 50 mm	52 (23.4)	21 (31.8)	3 (21.4)	2 (18.2)	
	Unknown	73 (32.9)	15 (22.7)	3 (21.4)	4 (36.4)	
5-Y survival*	%	46.5	75.0	50.0	11.1	0.001
10-Y survival**	%	32.0	64.7	37.5	0.0	<0.001

*142 osteosarcoma, NOS, 44 chondroblastic osteosarcoma, 10 fibroblastic osteosarcoma, 9 Paget osteosarcoma

**128 osteosarcoma, NOS, 34 chondroblastic osteosarcoma, 8 fibroblastic osteosarcoma, 9 Paget osteosarcoma

*mm* millimeters, *N* number of cases, *P p* value, *Y* year

## Discussion

Skull OSC and EWS are rare tumor entities. Using the SEER database we identified 321 skull OSC and 80 skull EWS cases and were able to show that the latter have a significantly better overall survival. Older age, tumor advancement, no surgical treatment, the use of radiotherapy, and early cases were independent predictors of poor survival in skull OSC. As in other tumor sites, skull EWS generally occurs at a younger age and osteosarcomas have a peak incidence around 20 years of age. Both tumors were generally resected, but radiotherapy is more commonly administered in EWS. In subtypes of OSC, chondroblastic OSC had a significantly better overall survival compared to osteoblastic OSC, while those arising in Paget’s disease of the bone have the most dismal prognosis.

### Osteosarcomas of the skull

Since its first description by Garland in 1945, literature has been filled with a little more than 150 skull OSC cases to date [18, 23]. An estimated 1.6% of all OSC arises from the skull [28]. They frequently present as a painless or only mildly tender lesion, while those arising in long bones are typically painful [23, 26, 40]. Other symptoms are dependent on their anatomical site [40]. They can include headache, cranial nerve palsy, exophthalmos, and visual impairment [17, 36]. Intracranial involvement has been reported in 14.1% of all skull OSC [42]. These cases all had dismal prognoses with less than 30% surviving more than 1 year [42].

If a bone sarcoma of the skull is suspected, infiltrative lesion identified on plain radiograph should be followed by an MRI to further characterize the tumor location, extension, and aspect [37]. Preferably, a core needle biopsy or open biopsy is performed [37]. If an OSC is diagnosed, patients should receive neoadjuvant chemotherapy, followed by resection of detectable disease and adjuvant chemotherapy [4, 15, 23, 37, 40, 42]. Currently, the combination of methotrexate, Adriamycin, and cisplatin has become standard of care [15, 37]. Radiotherapy may also be administered, but since OSC is relatively radiation-resistant, this may more commonly be reserved for inoperable or more advanced cases [40, 41]. The latter may partially explain the association of radiotherapy and overall survival in this study; however, only its association has been studied in this study. The effect of radiotherapy after resections with positive margins still remains to be investigated [21, 23]. Dural involvement is not uncommon and in cases with dural involvement, the dura needs to be resected [23, 29, 50]. Although wide surgical margins seem to improve survival [17, 36], this is commonly difficult to achieve because of adjacent critical structures [22, 50]. This study also showed a 31.5% GTR rate. Chemotherapy may improve outcomes by decreasing preoperative tumor size which might improve resection margins or management of residual tumor when GTR is not achievable [43].

This study found that Paget OSC has a significantly worse prognosis compared to osteoblastic OSC. In other sites of OSC, Paget-associated OSC also tend to occur in older people and have a more dismal prognosis [3, 24, 25]. The worse survival rate may partially be due to age, but stromal elements have also been shown to play a role in the malignant degeneration of bone, likely resulting in more aggressive disease [25]. The skull seems to be a preferential site for Paget OSC [36, 40]. While this study finds a 5-year survival rate of 51.0%, others reported 10–31.6% [9, 11, 42]. However, these often include early cases before the chemotherapy era. Metastasis of skull OSC is uncommon and less frequent compared to extracranial sites [22, 42]. Metastasis may occur to the lungs or brain; similar locations compared to extracranial OSC [8, 10, 42, 46]. In case of metastasis, survival is very poor [4, 26]. Recurrence is however more common, probably due to difficulty in achieving radical margins, especially in the skull base [10, 22, 23]. Recurrence is a poor prognosticator, commonly followed by death [10, 22].

### Ewing sarcomas of the skull

Since EWS arise in the skull in approximately only 1% of all cases, literature has been limited to case reports and case series. These EWS will most commonly present themselves with headaches and increased intracranial pressure, but scalp swelling can also be present [14, 39]. While typically EWS has an onion peel appearance, this is not always true in skull EWS [13, 39]. As in other sites of EWS, this study shows that skull EWS presents itself typically before 20 years of age [14, 20, 27, 39]. Metastatic disease at presentation is not common, with reports varying between 0 and 30% in series including all head and neck sites [19, 39]. This is in line with the low frequency in this study.

Standard-of-care in patients with Ewing sarcoma is neoadjuvant chemotherapy followed by local control with radiotherapy or wide excision of remaining tumor within reasonable limits of safety, or a combination [14, 19, 37, 39]. After local treatment, additional chemotherapy is administered to complete a total of 14 cycles [37]. In Europe vincristine, ifosfamide, doxorubicin, and etoposide are standard regimens; while in North America, a combination of vincristine, doxorubicin, cyclophosphamide, ifosfamide, and etoposide is administered [37]. In other series, radiotherapy is often administered, ranging from 54 to 100% in case series [14, 19, 30, 39]. This is also true for this series, where surgical treatment is frequent, commonly accompanied by radiotherapy. Some recommend that radiotherapy should always be administered, since microscopic free margins are next to impossible [14]. Dural involvement is nevertheless a common phenomenon in these tumors [39]. A recent study found that radiotherapy reduces local recurrences in limb EWS, but this effect was less clear in axial EWS [1]. Also, when wide resection was achieved, local control was not ameliorated [1]. One study in 51 EWS of the head and neck, including 32 of the skull, indicated that less than radical excision significantly decreases event-free and overall survival [19].

Older age is significantly associated with worse survival in EWS generally [5, 38]. However, many older patients present with larger, more advanced masses, and also more commonly in the pelvis; these are all three negative predictors of survival as well [5, 38]. In head and neck EWS, age over 15 has been reported to significantly reduce survival [19]. This was not found in this study, neither in univariate nor multivariate analyses. Also, in EWS tumors in other tumor sites, size over 150 cc and elevated LDH levels have been associated with worse survival [5]. In skull EWS, this has not yet been demonstrated. A five-year overall survival varies from 39 to 100% [14, 19, 30, 39]. With a 5-year survival rate of 68.5%, this study seems in line with previous findings. Survival is generally better compared to other sites of origin, which may partially be due to infrequent metastasis [19, 49]. Overall, 20–30% of extracranial EWS present with metastatic disease [5, 12]. Metastasis can occur in 75–80% of primary extracranial EWS within 2 years, most common sites are lungs and bones, but central nervous system involvement has been reported for 10–33% of cases [32, 33, 44]. Recurrence is relatively infrequent [14, 39]. In case of recurrence, death will usually follow shortly after [39]. Given the complexity of treatment in OSC and EWS of the skull, a multidisciplinary approach involving neurosurgeons, radiotherapists, and medical oncologists specialized in sarcomas is needed in order to optimize outcomes.

### Strengths and limitations

This study has several limitations, predominantly associated with the retrospective registry design. Many data of interest had a significant amount of missing values, such as tumor size, which had to be imputed using a validated algorithm. Unfortunately, the registry does not contain any information on recurrence and progression-free survival. Also, the mode and dosage of radiotherapy are not registered, nor is the indication of its use. This makes the interpretation of the exact impact radiotherapy has, adjacent to surgery, difficult and prone to confounding by indication. Since margin status is not registered in SEER, it may well be true that many patients receiving radiotherapy had positive margins which could explain the negative effect radiotherapy seems to have in OSC. Also, radiotherapy may have been used as salvage treatment. Furthermore, the use and regimen of chemotherapy could not be extracted either. However, since neoadjuvant chemotherapy has been common practice since the 1980s, we may assume that the larger part of these patients will have received systemic treatment. As treatment regimens have changed over the past decades, we have tried to compensate for these differences by modeling the year of diagnosis as well. As such, a significant difference in survival was seen in OSC during the pre-chemotherapy era, but was not observed in the EWS population, possibly due to the small amount of patients. Moreover, as the classification of sarcomas has changed over the last decades, there is a possibility that some of the included tumors would not be classified similarly nowadays. However, most of the tumors included in this study are treated after 1990. Lastly, the group of OSC NOS may possibly contain other histological subtypes than osteoblastic OSC. However, since other subtypes are registered separately and osteoblastic OSC are the most common subtype of OSC, we may assume that these are also the most prevalent as has previously been done [3]. Despite these limitations, the SEER database allows for investigation of rare tumor sites of OSC and EWS. To the best of our knowledge, this is the largest series described to date of both OSC and EWS arising from the skull. For the first time, it has been possible to stratify survival differences for four subtypes of skull OSC. These bone malignancies are not part of any neurosurgeon’s daily practice and are not necessarily part of centralized healthcare. Nationwide data provide a means to inform neurosurgeons on generally used therapeutic regimens and survival of these tumors in order to optimally inform and treat their patients.

## Conclusion

Both skull OSC and EWS generally benefit from surgery. Skull OSC has a significantly worse survival compared to EWS. Older age, tumor advancement, no surgical treatment, and the use of radiotherapy are independent predictors of poor survival in skull OSC. Chondroblastic OSC of the skull have a significantly better overall survival, while those arising in Paget’s disease of the bone have a dismal prognosis.
